# The Potential of IgG to Induce Murine and Human Thymic Maturation of IL-10+ B Cells (B10) Revealed in a Pilot Study

**DOI:** 10.3390/cells9102239

**Published:** 2020-10-05

**Authors:** Amanda Harumi Sabô Inoue, Aline Aparecida de Lima Lira, Marília Garcia de-Oliveira, Thamires Rodrigues de Sousa, Fábio da Ressureição Sgnotto, Alberto José da Silva Duarte, Jefferson Russo Victor

**Affiliations:** 1 Laboratory of Medical Investigation LIM 56, Division of Dermatology, Medical School, University of Sao Paulo, Sao Paulo 05403-000, Brazil; amanda_inoue@hotmail.com (A.H.S.I.); line_llira@hotmail.com (A.A.d.L.L.); mariliagarcia@usp.br (M.G.d.-O.); sousarthamires@gmail.com (T.R.d.S.); adjsduar@usp.br (A.J.d.S.D.); 2 Division of Hematology, Medical School, University of Sao Paulo, Sao Paulo 01246-903, Brazil; fabio.house@hotmail.com; 3 Division of Pathology, Medical School, University of Sao Paulo, Sao Paulo 01246-903, Brazil; 4 Division of Environmental Health, FMU, Laureate International Universities, Sao Paulo 04505-002, Brazil

**Keywords:** IgG, thymus, B cells, B10 cells, mouse, human

## Abstract

Regulatory B (B10) cells can control several inflammatory diseases, including allergies; however, the origin of peripheral B10 cells is not fully understood, and the involvement of primary lymphoid organs (PLOs) as a primary site of maturation is not known. Here, using a murine model of allergy inhibition mediated by maternal immunization with ovalbumin (OVA), we aimed to evaluate whether B10 cells can mature in the thymus and whether IgG can mediate this process. Female mice were immunized with OVA, and offspring thymus, bone marrow, spleen, lung, and serum samples were evaluated at different times and after passive transfer of purified IgG or thymocytes. A translational approach was implemented using human nonatopic thymus samples, nonatopic peripheral blood mononuclear cells (PBMCs), and IgG from atopic or nonatopic individuals. Based on the expression of CD1d on B cells during maturation stages, we suggest that B10 cells can also mature in the murine thymus. Murine thymic B10 cells can be induced in vitro and in vivo by IgG and be detected in the spleen and lungs in response to an allergen challenge. Like IgG from atopic individuals, human IgG from nonatopic individuals can induce B10 cells in the infant thymus and adult PBMCs. Our observations suggest that B10 cells may mature in the thymus and that this mechanism may be mediated by IgG in both humans and mice. These observations may support the future development of IgG-based immunoregulatory therapeutic strategies.

## 1. Introduction

The regulation of offspring allergy development mediated by maternal immune status has already been shown by several groups [1,2,3,4]. Some recent discussions and evidence have suggested that this mechanism involves the modulation of cell maturation in the offspring’s primary lymphoid organs (PLOs) [5,6], and the ability of maternal IgG to induce peripheral regulatory B cells (B10) in mice has been shown in murine peripheral cells [7,8]; however, the possible involvement of PLOs as a source of B10 cells has not been evaluated.

Approximately ten years ago, murine B10 cells were described as IL-10-producing B cells that express the constitutive molecule CD1d at high levels (CD1d^high^) and can control T cell-dependent inflammatory responses [9]. Shortly thereafter, murine B10 cells were described as being capable of reversing and preventing allergic inflammation [10,11]. B10 cells have subsequently been described as a regulatory population that play a pivotal role in inhibiting inflammation in several murine models [11,12,13,14,15,16] and participate in human immune regulation [17,18,19,20,21,22].

Together, these studies demonstrate the peripheral induction and functionality of B10 cells, but whether these cells can originate in PLOs or in which PLOs they can be generated has not been elucidated.

It was demonstrated in the early 2000s that the thymus contributes to peripheral pools of B cells as well as of alphabeta (αβ) and gammadelta (γδ) T cells [23]. This work showed that a significant number (approximately 3 × 10^4^ per day) of B220 + IgM+ mature B cells migrate from the thymus and that the thymus possesses substantial amounts of pro-B and pre-B cells.

In 2015, B10 cells were identified in a murine primary organ, the thymus, and the adoptive transfer of these cells revealed that they could mediate the suppression of an autoimmune response in lupus-like mice [24]. However, the maturation of B10 cells in the thymus remains elusive.

Here, using a standardized murine model of allergy inhibition mediated by maternal immunization, in which the induction of B10 cells was demonstrated [7,8], we aimed to investigate whether the thymus can mature B10 cells, and to generate evidence regarding the potential of maternal immunization to modulate this process with a translational approach.

## 2. Materials and Methods

### 2.1. Mice

C57BL/6 inbred wild-type (WT) male and female mice were used at 8–10 weeks of age. The animals were purchased from the Central Animal Facility of the School of Medicine and Institute of Biomedical Sciences, University of Sao Paulo (USP). The offspring (F1) of both sexes were evaluated during the neonatal period, and samples from at least three independent experiments were studied. All experiments described in this manuscript were approved by the University of Sao Paulo School of Medicine Animal Ethics Committee (CEP-ICBUSP: n-014, fl-028, l-03 - Sao Paulo, SP, Brazil).

### 2.2. Patient Samples

Thymus tissue was obtained from 14 patients who underwent corrective cardiac surgery at the Hospital do Coração (HCor) in Sao Paulo, Brazil. The evaluated patients exhibited no signs of immunodeficiency, genetic syndromes, or allergic reactions, and a patient age of less than seven days was used as an inclusion criterion (patient age, mean ± standard error (SE): 3.4 ± 0.54 days). Parental allergic backgrounds were evaluated, and only children with nonatopic mothers were included in this study.

Additionally, blood samples were collected from both male and female subjects ranging in age from 20 to 40 years who were previously classified as atopic or nonatopic. The classification conditions were as follows: (i) Atopic individuals: Clinically allergic as confirmed by medical consultation, with clinical symptom-related IgE-specific titers detected for at least two of the tested allergens in an immunoblot assay and reactivity to at least two of the tested allergens in a skin prick test (SPT); (ii) nonatopic individuals: Clinically nonallergic as confirmed by medical consultation, with no detectable IgE-specific titers for all tested allergens in an immunoblot assay and no reactivity to all tested allergens in an SPT. We excluded volunteers with severe eczema or dermographism or who used any of the following within 15 days of the test: Antihistamines, glucocorticosteroids, or other systemic drugs that could influence the SPT results.

Additional information about these individuals is shown in Table 1. Peripheral blood mononuclear cells (PBMCs) were collected from nonatopic volunteers. Each PBMC and thymus sample was provided by a different donor and analyzed in three independent experiments. The ethics committees at the HCor and the School of Medicine at USP approved this study (CAAE: 15507613.4.0000.0060).

### 2.3. SPT, Serum Antiallergen IgE Determination, and Collection of Blood Samples

SPTs were performed in accordance with European standards [25] using an adapted panel of allergens, including a profile of Brazilian allergens, as described in Table 1.

Serum-specific IgE antibodies were measured with a multiplex immunoblot assay according to the manufacturer’s instructions (EUROLINE Inhalation 2—EUROIMMUN AG, Lubek, Germany). The extracts that were tested are described in Supporting Table 1. Briefly, the strips were incubated with patient sera, washed, and incubated with alkaline phosphatase-conjugated anti-human IgE. After the second washing step, the strips were incubated with the chromogen/substrate solution. The reaction was stopped by washing, and the strips were evaluated with EUROLineScan software (EUROIMMUN, Lubek, Germany) to obtain semiquantitative results.

The band intensities were measured and converted into a score from zero to six, divided into the following concentrations (all expressed in kg/L): Class 0 < 0.35; class 1 < 0.7; class 2 < 3.5; class 3 < 17.5; class 4 < 50.0; class 5 < 100.0; and class 6 ≥ 100.0. According to the manufacturer’s instructions, individuals with a reactivity equal to or greater than class 2 frequently develop clinical symptoms; therefore, we considered reactivity to include only individuals with a reactivity equal to or above class 2 in our analyses.

After evaluating the SPT and antiallergen IgE determination results, two blood samples were obtained from each chosen volunteer via venipuncture and placed in tubes without anticoagulants. After the blood samples were centrifuged at 940× *g* for 10 min, the serum was fractionated, pooled, and stored at −80 °C.

### 2.4. Determination of Murine and Human Total IgG Subclasses

The levels of murine total IgG subclasses were measured by ELISA as previously described [8]. A standard curve was constructed to calculate the levels of each IgG subclass (Pharmingen, San Diego, CA, USA). Total human IgG subclasses were measured according to the specifications of the BINDARID Radial Immunodiffusion Kit (RID—Binding Site, Birmingham, UK) as previously described [26]. Ring diameters were measured, and the concentrations were determined using a reference table provided in the kit.

### 2.5. Murine Serum Total IgE Levels and Anaphylactic Anti-OVA IgE Titers

Total IgE antibodies were measured by ELISA as previously described [27]. To measure the total IgE level, a standard curve was used (Pharmingen, San Diego, CA, USA). The anaphylactic anti-OVA IgE titer was measured through passive cutaneous anaphylaxis (PCA) as previously described [28].

### 2.6. Murine Immunization

Female WT mice were immunized subcutaneously with 6 mg of alum (FURP, Sao Paulo, Brazil) alone or supplemented with 150 μg of OVA (EndoFit™—endotoxin levels <1 EU/mg; InvivoGen, San Diego, CA, USA). These animals were boosted intraperitoneally (i.p.) after 10 and 20 days with 100 μg of OVA in saline. The females that were immunized with alum only were boosted with saline alone. All females were mated 21 days postimmunization.

Some groups of offspring from the Alum/OVA-immunized and Alum-immunized mothers were immunized i.p. with 100 μg of OVA in 0.6 mg of alum at 3 days old (d.o.) and boosted after 10 days. Experimental analyses of the offspring were performed at 20 d.o. (Figure S1).

### 2.7. Passive In Vivo Transfer of Purified IgG

Normal female mice were subjected to passive prenatal transfer of Alum-immunized or Alum/OVA-immunized purified IgG as previously standardized by our group [26,28]. Females intravenously received 400 μg of purified IgG (totaling 1600 μg) at 10, 13, 17, and 20 days of gestation. The offspring from the mothers that received Alum-immunized or Alum/OVA-immunized IgG were evaluated at 3 d.o. (Figure S2).

### 2.8. Passive in vivo Transfer of Thymocytes

Normal 20-d.o. mice were subjected to passive transfer of thymocytes obtained from 20-d.o. offspring of Alum-immunized or Alum/OVA-immunized mothers. These mice received 3 × 10^7^ thymocytes from Alum-immunized or Alum/OVA-immunized mothers previously stained with succinimidyl ester (CFSE, CellTrace, Invitrogen, Waltham, MA, USA), and after two days, their spleens were evaluated by flow cytometry for the presence of CFSE+ B cells. Some groups of mice received 3 × 10^7^ thymocytes without staining and were subjected to the murine lung inflammation protocol (Figure S3).

### 2.9. Murine Lung Inflammation

The immunized offspring (3-d.o. immunization protocol) from either Alum-immunized or Alum/OVA-immunized mothers were immunized nasally with 100 μg of OVA (InvivoGen, San Diego, CA, USA) at 43, 50, 57, 58, and 59 d.o. The bronchoalveolar fluid (BAL) was analyzed at 60 d.o. following exsanguination via the abdominal aorta. The BAL was obtained by washing the lungs three times with 1.5 mL of PBS using a tracheal tube followed by centrifugation at 2000× *g* for 10 min. The cell pellet was diluted in 300 μL of PBS, and the total leukocyte counts were obtained using a Neubauer chamber. The lungs were surgically collected and subjected to a tissue dissociation protocol (Figure S1).

### 2.10. Purification of Mouse and Human IgG

IgG was purified from pooled serum in accordance with the Melon Gel IgG Spin Purification Kit protocol (Thermo, Waltham, MA, USA). Purified IgG was collected, sterilized using 0.20-micron filters (Corning, Berlin, Germany), and stored at -80 °C for use in cell culture experiments. IgG concentrations were determined using Coomassie Protein Assay Reagent (Pierce, Waltham, MA, USA) in accordance with the manufacturer’s instructions. The purity of IgG, as evaluated by SDS-PAGE, was greater than 95%. All pools were evaluated for the presence of IgA, IgM, and IgE antibodies, all of which were undetectable. Total murine IgG subclasses were measured by ELISA using standard proteins for each subclass, and total human IgG subclasses were measured using a BINDARID Radial Immunodiffusion Kit (RID—Binding Site, Birmingham, UK).

### 2.11. Spleen, Thymus, Bone Marrow and Lung Cell Suspensions

The spleen and thymus were collected aseptically, and cells were isolated for culture or flow cytometry analysis. To collect bone marrow cells, left and right femurs were harvested from 3-d.o. mice, stripped of periosteum, placed in petri dishes, cut at both ends, split open, and scraped gently with a needle to release the cells from the bone surface as well as bone marrow. The obtained cells (±1 × 10^6^ per mice) were placed in petri dishes with RPMI 1640 culture medium (Sigma, St. Louis, MO, USA).

Spleen, thymus, and bone marrow single-cell suspensions were prepared with cell strainers (BD Biosciences, Billerica, MA, USA) and placed in petri dishes containing RPMI medium. The cell suspensions were treated with lysis buffer (Biosource—ACK Lysis Buffer, Rockville, MD, USA) for 2 min and then washed twice with RPMI medium. The cells were subsequently resuspended in 1 mL of RPMI medium with 10% FetalClone III (FC-III—HyClone, Logan, UT, USA), and cell viability was quantified with 0.5% trypan blue in a Neubauer chamber.

Lung cells were collected by enzymatic dissociation with 1 mg/mL collagenase D and 2 mg/mL DNase I (Roche Diagnostics, Mannheim, Germany). Next, 5% FBS was added, and the samples were incubated for 15 min at 37 °C under continuous agitation. The digested tissues were homogenized gently and filtered through a plastic sieve with cell strainers (BD Biosciences, Billerica, MA, USA) to remove aggregates. The resulting cell suspensions were washed twice with RPMI medium (Gibco—Life Technologies, Grand Island, NY, USA) and then centrifuged at 500× *g* for 5 min.

All cell suspension analysis in this study were performed with individual samples.

### 2.12. Murine Cell Culture

To investigate the in vitro effect of purified maternal IgG on offspring B cells, thymocytes from 3-d.o. normal offspring were cultured for 120 h at a density of 3 × 10^6^ cells/mL in RPMI 1640 (Gibco—Life Technologies, Grand Island, NY, USA) supplemented with 10% FC-III (HyClone, Logan, UT, USA) in the presence of 100 μg/mL purified IgG from Alum/OVA-immunized or Alum-immunized mothers. To evaluate intracellular cytokine production, all culture wells were administered 10 mg/mL brefeldin A (Sigma-Aldrich, St. Louis, MO, USA) 24 h before flow cytometry analysis.

### 2.13. Murine Flow Cytometry

For surface staining, single-cell suspensions were prepared in flow cytometry buffer (PBS, 1% BSA). Anti-CD19, anti-CD5, anti-B220, anti-CD1d, anti-IgM, anti-CD4, anti-CD8 and anti-CD43 antibodies directly conjugated to Cy-Chrome, PE, APC, Horizon V450, PE-Texas Red, PerCP, PerCP-Cy5 or FITC (BD Biosciences, Billerica, MA, USA) were used at their optimal concentrations, as determined by titration experiments and according to the origin tissue.

We also evaluated spontaneous intracellular cytokine production using a previously described method [8]. To standardize intracellular cytokine detection, we used stimulation with 10 ng/mL phorbol 12-myristate 13-acetate (PMA) and 250 ng/mL ionomycin for 6 h as a positive control [29]. As a negative control, we used ex vivo stained cells. Positive controls induced 2- to 5-fold increases in the percentages of all evaluated cytokines on B cells with similar mean fluorescence intensity (MFI) levels. In the negative controls, the percentages of all evaluated cytokines on B cells were 0.4% or less; therefore, the MFI was not measured.

For intracellular staining, the cells were first surface-stained, fixed (PBS, 1% formaldehyde—Sigma), permeabilized (PBS, 0.5% saponin—Sigma), and subjected to intracellular staining with IL-10 (JES5-16E3). For the evaluation of intracellular IL-10, the cells were cultured for 24 h at 3 × 10^6^ cells/mL in RPMI 1640 (Gibco—Life Technologies, Grand Island, NY, USA) supplemented with 10% FC-III (HyClone, Logan, UT, USA) without stimulus in the presence of 10 mg/mL brefeldin A during the last 12 h (Sigma-Aldrich, St. Louis, MO, USA). After surface marker staining, the cells were fixed in 4% formaldehyde in PBS (Merck, Kenilworth, NJ, USA) and then subjected to intracellular staining. Instrument compensation was performed using microbeads coated with anti-rat/anti-hamster antibodies (CompBeads, BD Biosciences, Billerica, MA, USA) and their conjugated antibodies. Acquisition of 300,000 events per sample was performed in the lymphocyte quadrant (as determined by ratio size/granularity) on an LSRFortessa cytometer (BD Biosciences, Billerica, MA, USA), and analysis was performed using FlowJo software 10.1 (Tree Star, Ashland, OR, USA).

For all experiments, cell gating was based on the specific isotype control values as well as the fluorophore minus 1 (FMO) setting. Maturation stages of B cells were determined in the thymus by analyzing singlet lymphocytes expressing CD4-CD8-B220 + IgM-CD43 + (ProB cells), CD4-CD8-B220 + IgM-CD43- (PreB cells), or CD4-CD8-B220 + IgM + (mature B cells) phenotypes, and in bone marrow by analyzing singlet lymphocytes expressing B220 + IgM-CD43+ (ProB cells), B220 + IgM-CD43- (PreB cells), or B220 + IgM + (mature B cells) phenotypes. B10 cells were determined in the thymus by analyzing singlet viable double-negative (CD4-CD8-) lymphocytes expressing CD19 + CD5 + CD1d^high^ (Figures S1 and S2).

To measure IL-10 in supernatants, a mouse CBA kit (Cytometric Bead Assay, BD Biosciences, Billerica, MA, USA) was used in accordance with the manufacturer’s instructions. In brief, microspheres of different fluorophore intensities were sensitized with anti-IL-10 and incubated with either serum or supernatant to generate a standard curve or in the presence of capture antibodies against the same cytokine conjugated to PE for 2 h at room temperature. After washing with buffer supplied by the manufacturer, the microspheres were analyzed with an LSRFortessa cytometer (BD Biosciences, San Diego, CA, USA), and the levels were determined using CBA analysis software.

BAL was prepared in PBS, and the cells were stained as previously described [30] with optimal concentrations of the following monoclonal antibodies provided by BD Biosciences (San Diego, CA, USA): MHCII (2G9), CCR3 (101.111), CD11c (HL3), CD3 (145-2C11), and B220 (RA3-6B2). To prevent nonspecific binding to Fc receptors, a 2.4G2 blocking reagent (6 µg/mL) was added to the monoclonal antibody mix. Lymphocytes were identified as FSClo/SSClo cells expressing CD3 or B220, and B cells were distinguished from T cells via MHCII expression in the (B220/CD3)^+^ gate. Granulocytes were recognized as non-autofluorescent highly granular (SSChi) cells. Within this gate, eosinophils were defined as those that expressed the eotaxin receptor CCR3, intermediate levels of CD11c, and very low to undetectable levels of MHCII, B220, and CD3. Neutrophils presented a scatter profile similar to that of eosinophils but lacked CCR3 expression. Alveolar macrophages were identified as large autofluorescent cells.

### 2.14. Separation of Human Thymocyte Suspensions

Thymocyte separation was performed using Ficoll–Paque Plus (GE Healthcare, Danderyd, Sweden) after centrifugation as previously described [31]. Cell viability was assessed by flow cytometry.

### 2.15. Human Cell Culture

Suspensions of thymocytes were washed and resuspended in RPMI 1640 medium containing 10% FC-III (HyClone, Logan, UT, USA). A cell suspension aliquot was diluted in trypan blue (Sigma, USA) to evaluate the cell viability and number using a Neubauer chamber. Then, 1 × 10^6^ viable thymocytes were placed in each well of a 96-well culture plate (Costar, Cambridge, MA USA) and cultured with 100 μg/mL IgG purified from pooled serum samples from atopic or nonatopic individuals in RPMI 1640 medium containing 10% FC-III (HyClone, Logan, UT, USA). The mock condition or the addition of 100 μg/mL commercially purified IgG (IVIg) was used as a control. The culture plates were incubated for 3 or 7 days, and 1 µg/mL brefeldin A (Sigma, Tel-Aviv, Israel) was added in the last 12 h. Cell staining was performed to evaluate cell labeling via flow cytometry. For cell viability analysis, the cells were incubated with Live/Dead (PE-Texas red) fluorescent reagent (ThermoFisher, Waltham, MA, USA). All extracellular and intracellular analyses were performed using viable cells.

### 2.16. Human Flow Cytometry

To perform extracellular staining, 0.5 × 10^6^ cells/mL thymocytes were incubated with 1 μg of each antibody. Then, the samples were washed and fixed with formaldehyde. Thymocytes were stained with mouse anti-human CD4, CD8, and CD19 or isotype control antibodies (BD Pharmingen, Franklin Lakes, NJ, USA).

To perform intracellular labeling, 1 μg of each antibody was added to the cells in PBS containing 0.05% saponin. Thymocytes were stained with mouse anti-human IL-10 or an isotype control conjugated with the corresponding fluorophores (BD Pharmingen, Franklin Lakes, NJ, USA). Acquisition of 300,000 events per sample was performed in the lymphocyte quadrant (as determined by ratio size/granularity) on an LSRFortessa cytometer (BD Biosciences, San diego, CA, USA), and analysis was performed using FlowJo software 10.1 (Tree Star, Ashland, OR, USA). In thymus and PBMC samples, CD4-CD8-CD19+ cells were considered B cells.

### 2.17. Statistical Analysis

Statistical analysis was performed with GraphPad Prism 5.0 (GraphPad Software Inc., La Jolla, CA, USA). Data from in vivo and in vitro studies were taken from 3 to 5 separate experiments with 9 to 15 mice per group, 14 different thymus donors, or 14 atopic/nonatopic individuals, as indicated in the figure legends. Differences were considered significant at *p* ≤ 0.05 as assessed by Student’s *t*-test and the Mann–Whitney U test (comparisons between two groups) or by an ANOVA test (comparisons among more than two conditions).

## 3. Results

### 3.1. Identification of B10 Cells in the Thymus and Bone Marrow

**Figure 1:**
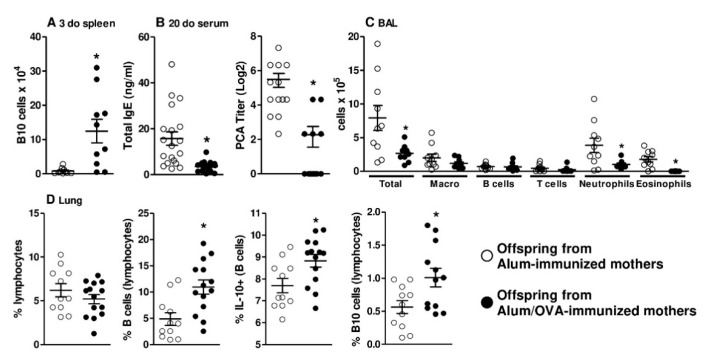
Effects of maternal immunization on offspring B10 cells, antibody production, and lung inflammation. The number of splenic B10 cells was evaluated in offspring from Alum-immunized (*n* = 10) or Alum/OVA-immunized (*n* = 10) mothers (**A**) Pups from both groups (*n* = 13–19 pups in each group; pups were delivered from four or five mothers in each group) were immunized with ovalbumin (OVA) in the neonatal period and evaluated at 20 days old (d.o). Total IgE levels were determined by ELISA, and anaphylactic IgE was determined by PCA (**B**) Bronchoalveolar fluid (BAL) preparation and dissociation of offspring lungs (*n* = 10–14 in each group) were performed on 60-d.o. offspring after five intranasal challenges. The differential cell counts in BAL were evaluated by flow cytometry (**C**). The dissociated lungs were evaluated by flow cytometry (**D**) Data are presented as each individual value, mean ± SEM. * *p* ≤ 0.05 compared with the Alum-immunized groups using Mann–Whitney U test.

**Figure 2:**
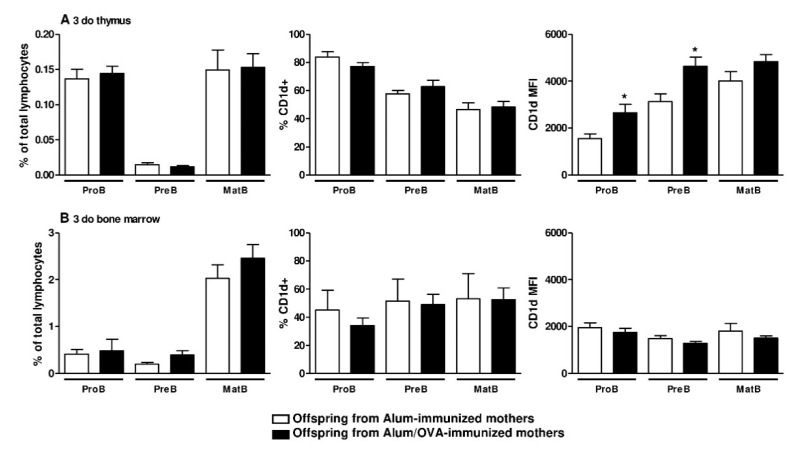
Identification of B cell precursors and CD1d expression evaluation in offspring bone marrow and thymic samples. Female mice were immunized with Alum or Alum/OVA, boosted 10 and 20 days later with saline or OVA, respectively, and mated at day 21. Offspring thymocytes and bone marrow from both groups were evaluated at 3 d.o. (*n* = 12–13 pups in each group; pups were delivered from four mothers in each group). Gating strategy to identify thymic and bone marrow B cell maturation stages and CD1d expression are demonstrated in Figure S4. (**A**) Total thymic B cell maturation stages (ProB, PreB, and MatB) are expressed as the percentage of cells gated as lymphocytes, the CD1d frequency, and intensity of expression at each maturation stage were evaluated. (**B**) Total bone marrow B cell maturation stages (ProB, PreB, and MatB) are expressed as the percentage of cells gated as lymphocytes, the CD1d frequency and intensity of expression at each maturation stage were evaluated. Data are presented as the mean ± SEM. * *p* ≤ 0.05 compared with the Alum-immunized groups using Mann–Whitney U test.

First, we analyzed some main parameters of the offspring to ensure that the chosen murine model was adequate for the proposed work. As shown in Figure 1A–D, compared to offspring from mothers immunized with only the adjuvant used for OVA immunization (alum-immunized), offspring from mothers immunized with OVA (Alum/OVA-immunized) exhibited the following characteristics: Augmented peripheral (splenic) B10 cells; inhibited total and specific anaphylactic IgE production; inhibited total cell infiltrate (mainly neutrophils and eosinophils) in the lungs after an antigen challenge; and augmented total B cells, IL-10+ B cells, and B10 cells (CD5+ CD1d^high^) in the lungs after an antigen challenge. In this murine model, we analyzed the phenotype of B cells during their maturation process in the two main PLOs, the thymus and the bone marrow, during the neonatal period. Maternal OVA immunization did not influence the frequency of B cell detection at each main maturation stage (ProB, PreB, and Mature IgM+) in the offspring thymus (Figure 2A) and bone marrow (Figure 2B) relative to that in offspring from Alum-immunized mothers.

**Figure 3:**
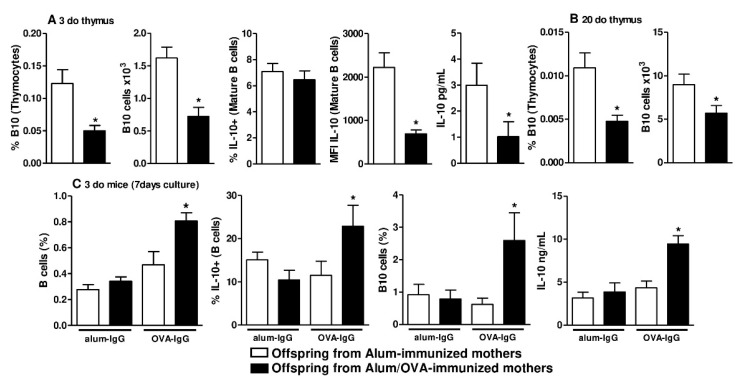
Frequency and number of offspring thymic B10 cells in vivo and in response to in vitro IgG stimuli. Female mice were immunized with Alum or Alum/OVA, boosted 10 and 20 days later with saline or OVA, respectively, and mated at day 21. The frequency and number of thymic B10 cells in 3-d.o. offspring (*n* = 12–13 pups in each group; pups were delivered from four mothers in each group) were evaluated (**A**) We also evaluated the frequency and intensity of IL-10 expression on mature B cells and spontaneous IL-10 secretion in the supernatant of a 24-h culture of total thymocytes. The frequency and number of thymic B10 cells were also evaluated in 20-d.o. offspring (**B**) IgG antibodies were purified from Alum-immunized (*n* = 25) or Alum/OVA-immunized (*n* = 25) females. Normal offspring thymocytes (*n* = 10) were cultured for 7 days with 100 μg of purified IgG from Alum-immunized (alum-IgG) or Alum/OVA-immunized (OVA-IgG) mothers (**C**) Cultures were performed with total thymocytes and total B cells, and IL-10 expression and the frequency of B10 cells were evaluated by flow cytometry. Data are presented as the mean ± SEM. * *p* ≤ 0.05 compared with the Alum-immunized groups using Mann–Whitney U test.

Although B10 cell phenotypes during the maturation process of B cells in PLOs have not been described, these cells can be identified by their high expression of CD1d at maturity (CD1d^high^); therefore, we evaluated the frequency and intensity of CD1d expression at different B cell maturation stages (Figure S4), revealing that maternal OVA immunization did not influence the frequency of CD1d expression in either evaluated organ (Figure 2A,B). By evaluating the intensity of CD1d expression, as determined by MFI measurement, we observed that maternal OVA immunization induced higher expression of the CD1d molecule at the ProB and PreB stages of B cell maturation in the offspring thymus (Figure 2A) than adjuvant immunization. This effect was not observed in the offspring bone marrow (Figure 2B). We next evaluated the frequency of B10 cells in the offspring thymus and found a reduced frequency and absolute number of mature B10 cells (CD19 + CD5 + CD1d^high^) in offspring from Alum/OVA-immunized mothers relative to offspring from Alum-immunized mothers (Figure 3A and Figure S5). In the same group of offspring, we also observed that neonatal thymic B cells had a similar frequency but a lower intensity and lower secretion levels of IL-10 after 24 h of culture (Figure 3A and Figure S6). The reduced frequency and number of B10 cells persisted in 20-d.o. offspring (Figure 3B and Figure S7).

### 3.2. Functional and In Vivo Evidence of Thymic B10 Cells

To elucidate whether the thymus can generate B10 cells, we adopted an experimental protocol to induce murine peripheral B10 cells using purified IgG [7,8]. To this end, we cultured offspring thymocytes from both groups with purified IgG from OVA (OVA-IgG)- or alum (alum-IgG)-immunized females. The frequency of IgG subclasses in both purified IgG pools was similar between the alum-IgG and OVA-IgG groups (Figure S8A)**.** After 7 days of culture, a period that allowed for cell maturation, thymocytes from the offspring of Alum/OVA-immunized mothers had a higher frequency of B cells after culture with OVA-IgG than those from the offspring of Alum-immunized mothers, and no effect was observed in response to alum-IgG (Figure 3C). As a result of specific OVA-IgG stimulation, thymocytes from the offspring of Alum/OVA-immunized mothers produced IL-10 at a higher frequency, demonstrating an augmentation of B10 cell numbers under these conditions and higher secretion levels of IL-10 (Figure 3C).

**Figure 4:**
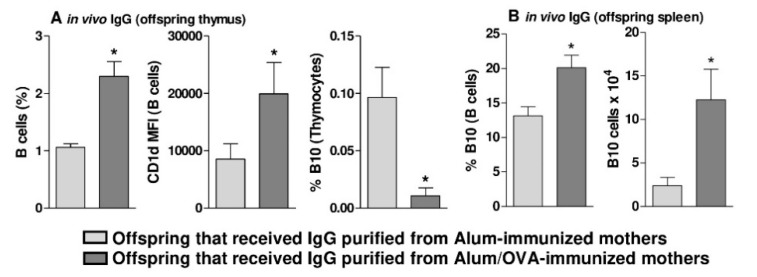
Frequency and number of offspring thymic B10 cells in response to maternal passive treatment with IgG. During gestation, normal females passively received 1600 μg of purified IgG (*n* = 5 females per group), and the frequency of total B cells, the intensity of CD1d expression, and the frequency of B10 cells in the thymus of 3-d.o. offspring (*n* = 12–13 pups in each group; pups were delivered from five mothers in each group) were evaluated (**A**) We also assessed the frequency and number of B10 cells in the spleens of offspring in the same period and groups (**B**) Data are presented as the mean ± SEM. * *p* ≤ 0.05 compared with offspring that received IgG from the Alum-immunized mothers using Mann–Whitney U test.

Next, we performed an in vivo experiment in which both purified IgG pools were passively transferred to females during pregnancy at a dose that was determined based on previous experiments by our group [26,28]. This experiment revealed that the passive transfer of OVA-IgG induced a higher frequency of total B cells (including ProB, PreB, and MatB cells) expressing CD1d at a high intensity but a reduced frequency of mature B10 cells (CD5 + IL-10 + CD1d^high^) in the offspring thymus (Figure 4A). However, evaluation of the spleens of these same animals showed that in vivo treatment with OVA-IgG led to an augmented frequency and number of peripheral B10 cells (CD5 + IL-10 + CD1d^high^) relative to that achieved with alum-IgG treatment (Figure 4B), revealing a peripheral profile very similar to that of offspring derived from Alum/OVA-immunized females.

### 3.3. In Vivo Evidence that Thymic B10 Cells Can Migrate to the Spleen And Inflammatory Sites

To further evaluate thymic B10 cell migration, we performed passive transfer of thymocytes derived from alum- or Alum/OVA-immunized offspring to normal mice.

**Figure 5:**
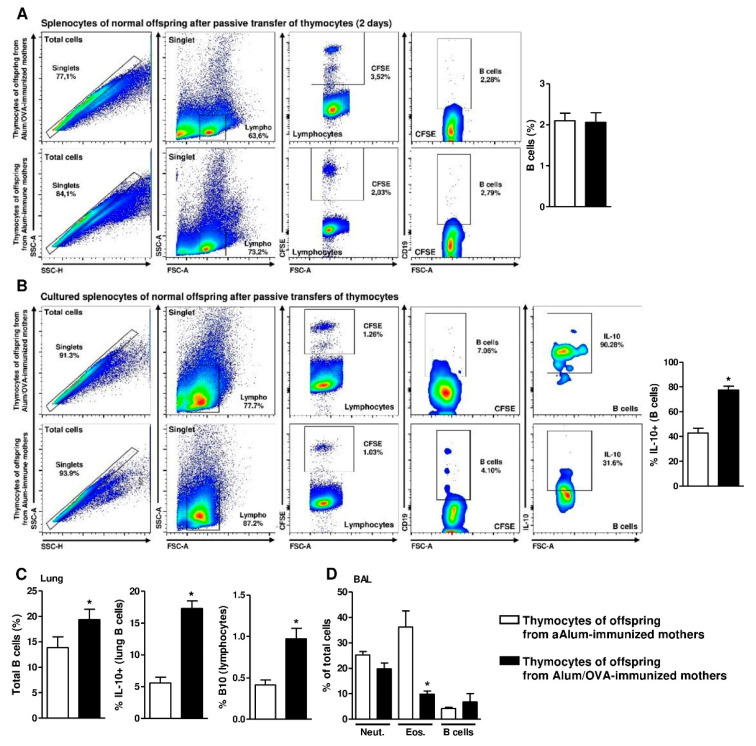
Identification and effect of passive transfer of thymocytes on offspring allergic inflammation. Some offspring from nonimmune mothers were passively administered total thymocytes from 20-d.o. offspring of Alum-immunized or Alum/OVA-immunized mothers (*n* = 8-9 pups in each group; pups were delivered from three mothers in each group). Twenty-d.o. offspring that passively received total CFSE-stained thymocytes were evaluated after 2 days, and the frequency of B cell transfer in the spleen is illustrated for both groups (**A**) Splenocytes from both groups were also cultivated without stimulus for 24 h, and the frequency of IL-10 production by transferred B cells is illustrated (**B**) Some groups of thymocyte-transferred mice were subjected to the allergic lung inflammation protocol, and the frequencies of B cells and IL-10-producing B cells were evaluated in lung tissue (**C**) BAL from the same groups was also evaluated, and the frequencies of neutrophils, eosinophils, and B cells are shown (**D**) Data are presented as the mean ± SEM. * *p* ≤ 0.05 compared with the Alum-immunized groups using Mann–Whitney U test.

Analysis of the normal recipients’ spleens showed similar frequencies of B cells from both donors after two days (Figure 5A). We then cultured those splenocytes and evaluated the production of IL-10 by the transferred B cells (CFSE+). We observed higher IL-10 production among the transferred splenic B cells derived from offspring of Alum/OVA-immunized mothers compared to that among cells derived from offspring of Alum-immunized mothers (Figure 5B).

Next, mice with thymocytes transferred from both groups were immunized with OVA and subjected to the lung inflammation protocol. Due to the duration of this protocol, the transferred B cells could not be evaluated; however, analysis of total B cells in the lung tissue revealed higher frequencies of total B cells, IL-10+ B cells and B10 cells (CD5 + IL-10 + CD1d^high^) in animals that received thymocytes from Alum/OVA-immunized offspring (Figure 5C). Evaluation of the BAL from these groups also revealed reduced eosinophil infiltration and a possible increase in B cell numbers (Figure 5D).

### 3.4. Human Nonatopic Infant Thymic and Adult Peripheral B Cells Can be Modulated to Produce IL-10 by Treatment with Purified IgG

Due to observations of murine thymic B10 cells, we utilized a translational approach to evaluate possible similarities in the human thymus. To do this, we first prepared purified IgG pools analogous to the preparations used in the murine model and IgG from allergic (derived from Alum-immunized mothers) and tolerant (derived from Alum/OVA-immunized mothers) animals. Thus, IgG was obtained from allergic (atopic) individuals and tolerant (nonatopic) individuals, and we first confirmed that the frequencies of IgG subclasses in both purified IgG pools were similar (Figure S8B).

**Figure 6:**
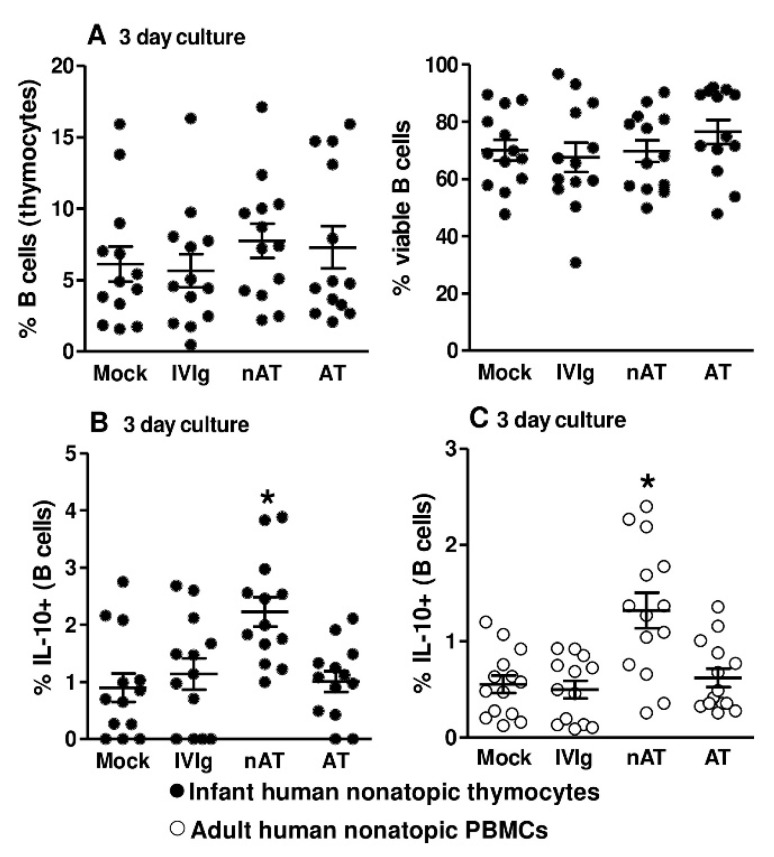
Effects of purified IgG on nonatopic infant intrathymic or nonatopic adult peripheral B cells. Purified IgG from atopic or nonatopic adults was pooled. Thymocytes from nonatopic infants younger than 7 d.o. (*n* = 13) were cultured in the absence (mock) or presence of 100 µg/mL commercial IgG (IVIg) or IgG purified from nonatopic (nAT) or atopic (AT) individuals. The frequency and viability of cultured B cells were evaluated after 3 days of culture (**A**) Spontaneous IL-10 production by thymic B cells was also evaluated (**B**) Freshly isolated PBMCs from nonatopic adults (*n* = 13) were cultured under the same conditions and evaluated after 3 days of culture. Spontaneous IL-10 production by peripheral B cells was also evaluated (**C**) Data are presented as each individual value, mean ± SEM. * *p* ≤ 0.05 compared with the control groups (mock and IVIg).

Next, human thymocytes from nonatopic newborns (delivered by mothers without an allergic background) were cultured with purified IgG pools from atopic or nonatopic adults; mock and IVIg conditions were used as controls. The frequencies and viabilities of B cells were similar in all conditions after 3 days of culture (Figure 6A). IL-10 production was detected at higher levels in response to the presence of nonatopic IgG relative to all other conditions after 3 days of culture (Figure 6B). We also evaluated the same parameters in freshly isolated peripheral blood mononuclear cells (PBMCs) from nonatopic adults. The frequencies and viabilities of B cells were also similar among all conditions after 3 days of culture (data not shown), and IL-10 production was detected at higher levels in response to the presence of nonatopic IgG relative to all other conditions after 3 days of culture (Figure 6C).

## 4. Discussion

Our initial results corroborate previous observations revealing that the murine model of maternal OVA immunization can inhibit offspring allergy development [27,28,32,33,34,35,36,37], as evidenced mainly by inhibition of the IgE response and lung inflammation in offspring derived from Alum/OVA-immunized mothers. Several mechanisms have been suggested to mediate the inhibitory effect of maternal immunization on offspring allergy development, including the induction of B10 cells [8].

Although B10 cells have been described in several murine models and human diseases [14,16,18,21,38], they were previously shown to be major players in the regulation of the allergic response in a murine model of OVA-induced lung inflammation [10,39].

The origin of B10 in mammals is poorly understood since B cells can also mature in the thymus [23]; therefore, we investigated tissues that are considered to be the main producers of B cells, i.e., the bone marrow and thymus. Phenotypes associated with mature B10 cells are diversified in the literature, but the most frequent and well established is CD19 + CD5 + CD1d^high^ cells [9,16]. The ontogeny of B10 cells is not described in the literature, and considering that high CD1d (CD1d^high^) expression is a main phenotypic characteristic of mature B10 cells, we evaluated the intensity of CD1d expression on immature B cells.

The presence of B10 cells in the murine thymus and their critical role in immune homeostasis were described in 2015 [24], but the maturation of these cells in the thymus was not evaluated. We analyzed the number of mature B10 cells in the offspring thymus and found upregulated CD1d expression on B cells during maturation stages in the thymic samples from offspring of Alum/OVA-immunized mothers and a lower frequency and number of B10 cells until 20 d.o., which is a controversial result. Given this result, we tested the described protocol for inducing peripheral B10 cells using purified maternal IgG [7,8] to determine if B10 cells can be induced in the thymus.

Our results demonstrate that thymic B cells can indeed be induced in response to maternal anti-OVA-IgG in vitro and that in vivo passive transfer of anti-OVA-IgG can induce a profile of thymic B cells very similar to that observed in response to maternal OVA immunization and contribute to the augmentation of peripheral B10 cells. This last observation suggested that the reduced number of mature thymic B10 cells in offspring from immunized mothers may be a consequence of preferential migration, apoptosis, or even additional factors concerning the ontogeny of B10 cells that are still unknown.

Next, we observed augmentation of peripheral B10 cells in offspring from anti-OVA-IgG-transferred mothers, which corroborates the hypothesis that anti-OVA-IgG augments mature thymic B10 cells that preferentially migrate from the thymus but does not eliminate the alternative hypothesis mentioned above and does not allow us to state that peripheral B10 cells have a thymic origin or even that the thymus can indeed mature B10 in physiological conditions. In this context, we must also consider that B10 cell maturation has also been suggested to occur in peripheral organs, including the spleen, and our observation cannot exclude the possible maturation of B10 cells in secondary lymphoid organs [9,40]. Here, we focused on identifying the role of PLOs as B10 cell sources, but more complex experiments must be undertaken to elucidate additional associated mechanisms. Together, these results suggest that the thymus may also serve as a site of B10 cell maturation; however, this organ has not yet been shown to be the main source of peripheral B10 cells.

Studies have already demonstrated that thymic B cells can participate in thymic Treg cell maturation [41,42] and that the regulatory function of peripheral B10 cells is related to Treg cells in murine models of allergy [10] and arthritis [12]. A recent study performed in the same allergy-induction murine model adopted in the present study demonstrated that mice from Alum/OVA-immunized mothers had a reduced frequency of thymic Tregs without any effects on the frequencies of peripheral and lung-infiltrated Tregs, and without consequential effects on the allergy inhibition observed in those animals [34]. The results obtained here corroborate these observations, suggesting that B10 cells can mature in the thymus and can be a possible cause of the described reduction in Treg frequency.

To generate some functional evidence about the activity of thymic B10 cells, we adoptively transferred thymocytes and found that thymic B10 cells could migrate to the spleen and secrete IL-10 at a high frequency. Furthermore, these animals were subjected to an allergy-induction protocol, and we found reduced lung inflammation with a higher frequency of infiltrating B10 cells. The role of B10 cells in inhibiting allergic inflammation and the mediation of this effect by IL-10 secretion has been extensively demonstrated and discussed in the literature since 2010 [39,43]. Due to technical limitations in obtaining an adequate number of purified B cells from the 3-d.o. offspring thymus to carry out a functional cell transfer protocol, our experimental protocol was performed using total thymocytes. Considering this last observation, we cannot state that thymic B10 cells are the main regulators of the murine allergic immune response in our murine model; however, we yield some evidence that thymic B10 cells are also candidates to exert immunoregulatory effects on allergic lung inflammation.

B10 cells have also been demonstrated to play a pivotal role in the control of inflammatory human diseases. The number of peripheral B10 cells is reportedly reduced in patients with rheumatoid arthritis [18] and increased during long-term remission after IgG treatment for pemphigus [38]. Other results have led some authors to suggest that autoimmune diseases be treated with autologous B10 cells [44]. Due to the similarities between our murine observations and those human observations, we decided to utilize a translational approach.

Using normal infant thymocytes and IgG from atopic or nonatopic individuals, we found that IgG from nonatopic (tolerant) individuals could efficiently induce thymic B cells to produce IL-10 when compared to IgG from atopic (allergic) individuals. This finding suggests that human thymic B cells have a similar potential to yield mature B10 cells in response to the donor’s IgG repertoire, as observed in our murine model, since the immune status of allergy tolerance is related to murine OVA-IgG.

Extensive literature evidence demonstrates that the immune status of IgG donors may be related to differential modulation of thymic lymphocytes [26,31,45,46], which has been discussed in the literature since 2014 [6,47]. Among related observations, nonatopic IgG may be related to thymic maturation of lymphocytes with an allergy inhibition-related cytokine profile induced by γδT cells [48] as well as T CD4+ and CD8+ cells [5]. The mechanism mediating these differential effects of IgG observed between different IgG repertoires is not understood, but a very recent hypothesis suggests that such effects may be due to variations in the IgG idiotype sets determined by environmental exposure; this hypothesis is called the “hooks without bait” theory [49].

Due to the difficulty of obtaining evidence of thymic lymphocyte migration in humans, we generated some evidence for the potential of human thymic B cells to become circulating B cells with regulatory potential using nonatopic adult peripheral B cells in a similar in vitro approach. These experiments revealed IL-10 induction in response to nonatopic IgG, demonstrating that similar to thymic B cells, human peripheral B cells can produce IL-10 in response to IgG.

At this point, we are still far from certain that human peripheral B10 cells are also generated in the thymus, as suggested in the murine model, but our translational observations demonstrated similarities between murine and human B10 cells, which has rarely been reported in the literature.

## 5. Conclusions

In conclusion, our observations suggest that the thymus may be an additional site of B10 cell maturation in mice and humans. Furthermore, in both species, IgG may play a role in the induction of B10 cells. Our work had some technical limitations, mainly due to the low frequency of B cells in the thymus. Thus, we could not elucidate the mechanism by which IgG mediates the induction of B10 cells in the thymus or demonstrate that thymic B10 cells can effectively regulate the immune response. Nevertheless, our evidence has high potential to initiate a new avenue of research to clarify this mechanism.

## Figures and Tables

**Table 1:** Characteristics of the individuals included in the study.

	Nonatopic	Atopic	*p*
Number	17	18	
Age, years (mean ± SE)	30.2 ± 4.11	32.9 ± 3.26	0.87
Sex (male/female)	7/10	9/9	0.82
**IgE-specific reactivity (*n*/%)**			
*Dermatophagoides pteronyssinus*	0/0	18/100	
*Dermatophagoides farinae*	0/0	18/100	
*Aspergillus fumigatus*	0/0	9/50	
*Penicillium notatum*	0/0	4/22	
*Alternaria alternata*	0/0	3/16	
*Canis familiaris*	0/0	2/11	
*Felis domesticus*	0/0	2/11	
*Cladosporium herbarum*	0/0	0/0	
**SPT reactivity (*n*/%)**			
*Dermatophagoides pteronyssinus*	0/0	17/93	
*Dermatophagoides farinae*	0/0	16/88	
*Aspergillus fumigatus*	0/0	6/33	
*Penicillium notatum*	0/0	3/16	
*Alternaria alternata*	0/0	2/11	
*Canis familiaris*	0/0	2/11	
*Felis domesticus*	0/0	1/5	
*Cladosporium herbarum*	0/0	1/5	
**SPT reactivity (*n*/%)**			
Dual	0/0	0/0	
Triple	0/0	9/50	
Quadruple	0/0	6/33	
Quintuple	0/0	3/16	
**Others**			
Clinically allergic	0/0	18/100	
Regular use of antihistamines	0/0	18/100	

SPT–skin prick test; mo—months.

## Supplementary Materials

The following are available online at https://www.mdpi.com/2073-4409/9/10/2239/s1: Figure S1. Schematic representation of the maternal immunization, offspring evaluation, offspring immunization, and murine lung inflammation protocols; Figure S2. Schematic representation of the passive in vivo transfer of purified IgG protocol; Figure S3. Schematic representation of the passive in vivo transfer of thymocytes protocols; Figure S4. Gating strategy to identify B cell precursors and evaluate CD1d expression in offspring bone marrow and thymic samples; Figure S5. Gating strategy to identify 3-d.o. offspring thymus B10 cells; Figure S6. Gating strategy to identify offspring thymus IL-10-producing B cells; Figure S7. Gating strategy to identify 20-d.o. offspring thymus B10 cells; Figure S8. Murine and human IgG subclasses.
