# Supplementary material for: The Potential of IgG to Induce Murine and Human Thymic Maturation of IL-10+ B Cells (B10) Revealed in a Pilot Study
Source: Cells. 2020 Oct 5;9(10):2239. doi: 10.3390/cells9102239 (PMC7600151; doi:10.3390/cells9102239)
Supplement: Supplementary file 1 [file cells-09-02239-s001.pdf]

Article

The potential of IgG to induce murine and human thymic maturation of IL-10+ B cells (B10) revealed in a pilot study

Amanda Harumi Sabô Inoue, Aline Aparecida de Lima Lira, Marília Garcia de Oliveira, Thamires Rodrigues de Sousa, Fábio da Ressureição Sgnotto, Alberto José da Silva Duarte, and Jefferson Russo Victor

Figure S1

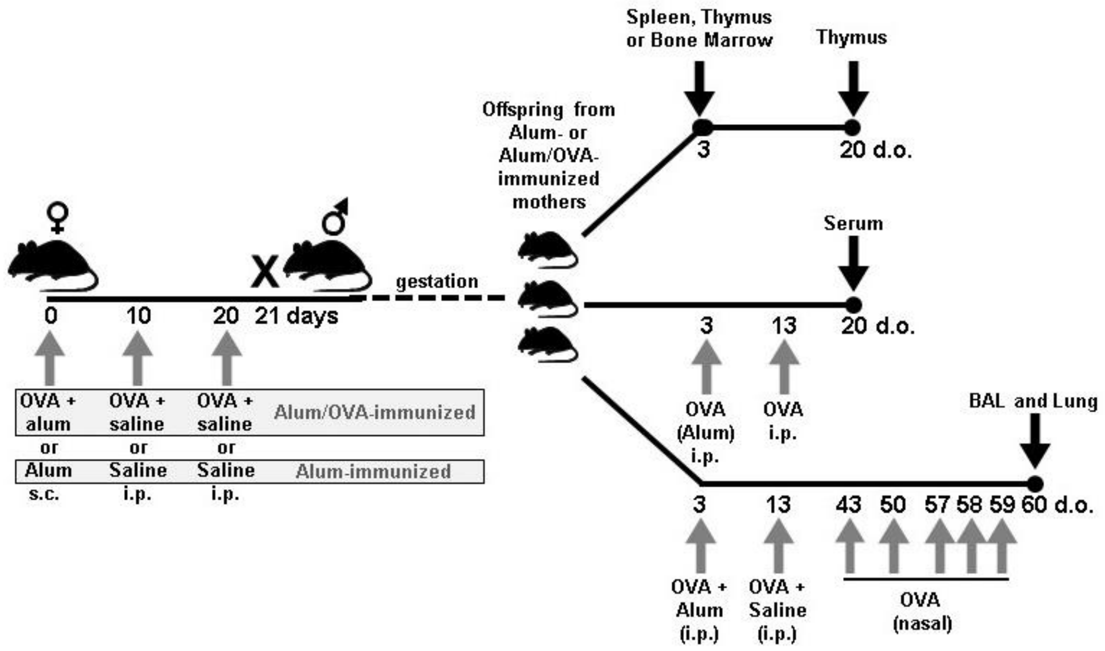

Figure S1. Schematic representation of the maternal immunization, offspring evaluation, offspring immunization and murine lung inflammation protocols. OVA= Ovalbumim; s.c.= subcutaneously; i;.p.= intraperitoneally; d.o.= days old; BAL= bronchoalveolar fluid.

Figure S2

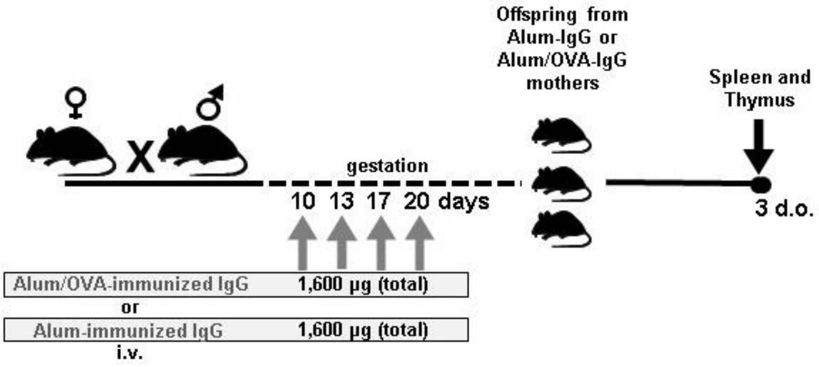

Figure S2. Schematic representation of the passive *in vivo* transfer of purified IgG protocol. OVA= Ovalbumim; i.v.= intravenously; d.o.= days old.

Figure S3

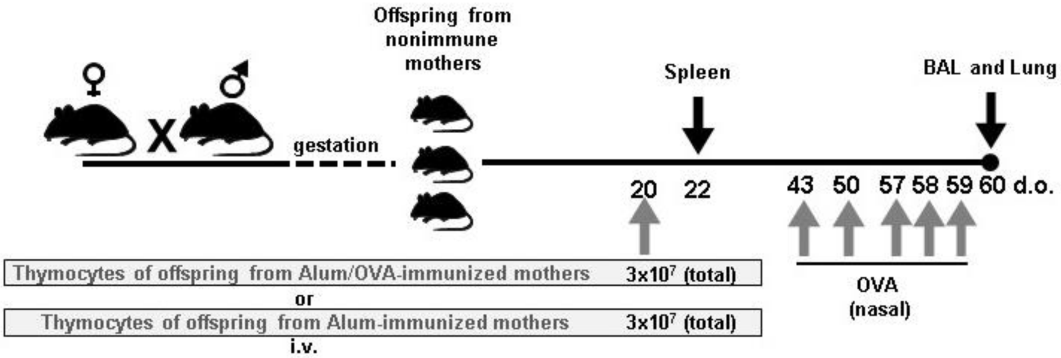

Figure S3. Schematic representation of the Passive in vivo transfer of thymocytes protocols. OVA= Ovalbumim; d.o.= days old; BAL= bronchoalveolar fluid.

Figure S4

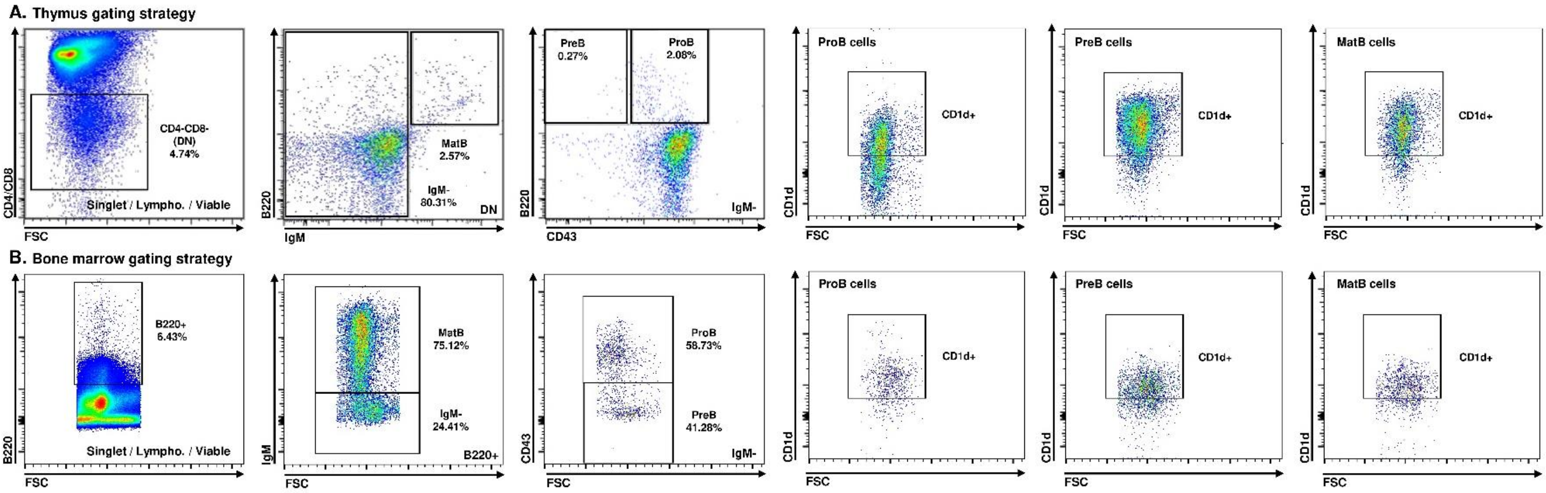

Figure S4. Identification of B cell precursors and CD1d expression evaluation in offspring bone marrow and thymic samples. Offspring thymocytes and bone marrow from both groups were evaluated at 3 d.o.. Each sample was acquired using a single-cell gate (determined by SSC-A/SSC-H parameters), a viable-cell gate (determined by LIVE/DEAD staining) and a lymphocyte gate (determined by relative SSC-A/FSC-A). For the thymus (A), double-negative (DN) cells (CD4-CD8-) were gated as B220+IgM+ to determine mature B cells (MatB), IgM- cells were gated, and the expression of CD43 was evaluated to determine ProB cells (CD43+) or PreB cells (CD43-). For bone marrow (B), B220+-gated cells were evaluated, IgM+ cells were gated to determine mature B cells (MatB), IgM- cells were gated, and the expression of CD43 was evaluated to determine ProB cells (CD43+) or PreB cells (CD43-).

Figure S5

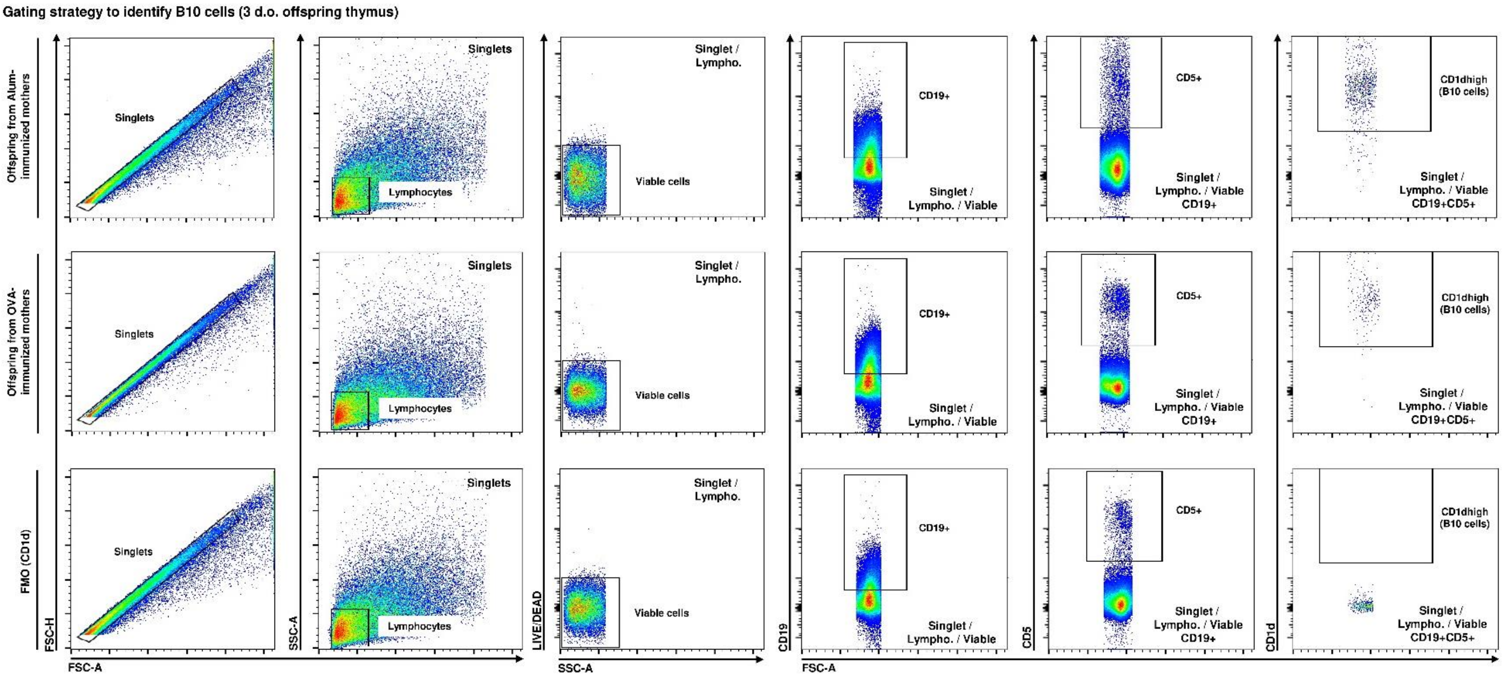

Figure S5. Gating strategy to identify 3 d.o. offspring thymus B10 cells. Each sample was acquired using a single-cell (Singlet) gate (determined by SSC-A/SSC-H parameters), a lymphocyte gate (determined by relative SSC-A/FSC-A). and a viable-cell gate (determined by LIVE/DEAD staining). Upper and middle panels illustrate the gating strategy to identify B10 cells on each group (Alum-immunized or OVA-immunized offspring). CD19+ cells were gated to determine B cells, and CD5 expression was evaluated on CD19+ cells. The expression of CD1d was evaluated on CD19+CD5+ cells, and CD1d^{high} cells were gated (as determined by FMO – lower panels) to identify B10 cells (Singlet+Lymphocytes+LIVE/DEAD-CD19+CD5+CD1d^{high}).

Figure S6

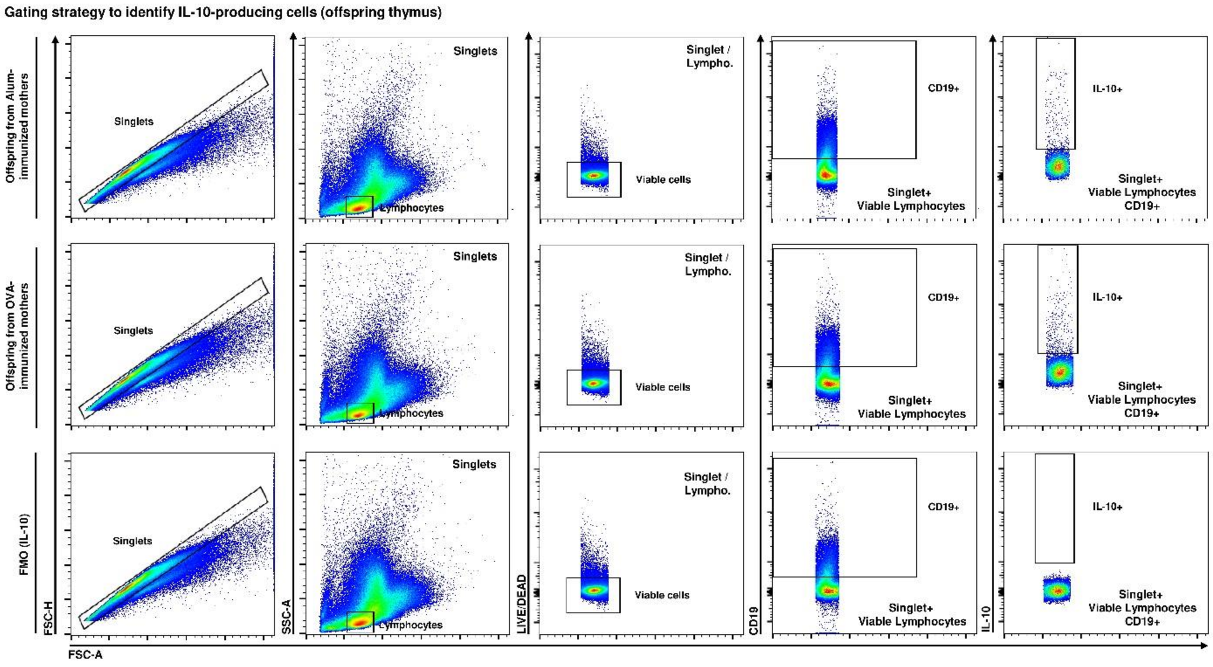

Figure S6. Gating strategy to identify offspring thymus IL-10-producing B cells. Each sample was acquired using a single-cell (Singlet) gate (determined by SSC-A/SSC-H parameters), a lymphocyte gate (determined by relative SSC-A/FSC-A). and a viable-cell gate (determined by LIVE/DEAD staining). Upper and middle panels illustrate the gating strategy to identify IL-10-producing B cells on each group (Alum-immunized or OVA-immunized offspring). CD19+ cells were gated to determine B cells, and IL-10 expression was evaluated on CD19+ cells. IL-10+ cells were gated (as determined by FMO – lower panels) to identify IL-10-producing B cells (Singlet+Lymphocytes+LIVE/DEAD-CD19+IL-10+).

Figure S7

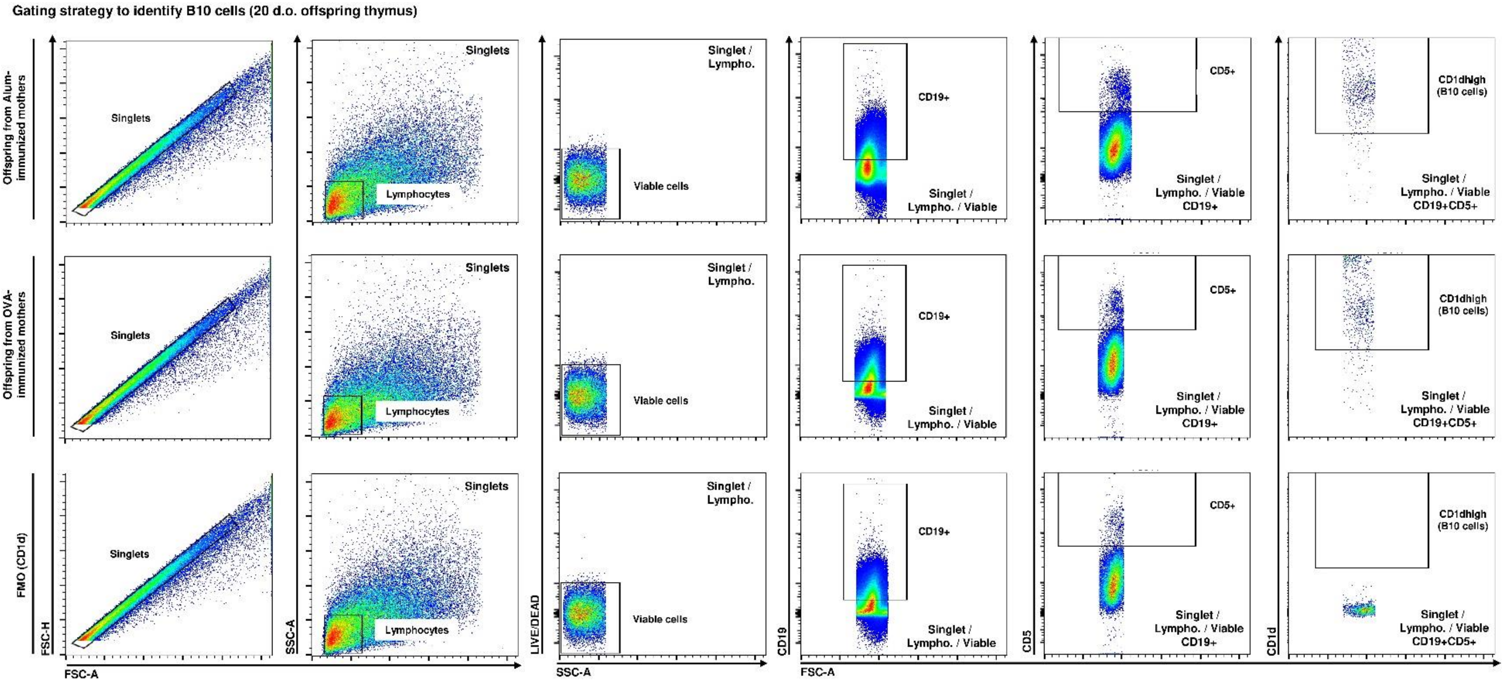

Figure S7. Gating strategy to identify 20 d.o. offspring thymus B10 cells. Each sample was acquired using a single-cell (Singlet) gate (determined by SSC-A/SSC-H parameters), a lymphocyte gate (determined by relative SSC-A/FSC-A). and a viable-cell gate (determined by LIVE/DEAD staining). Upper and middle panels illustrate the gating strategy to identify B10 cells on each group (Alum-immunized or OVA-immunized offspring). CD19+ cells were gated to determine B cells, and CD5 expression was evaluated on CD19+ cells. The expression of CD1d was evaluated on CD19+CD5+ cells, and CD1d^{high} cells were gated (as determined by FMO – lower panels) to identify B10 cells (Singlet+Lymphocytes+LIVE/DEAD-CD19+CD5+CD1d^{high}).

Figure S8

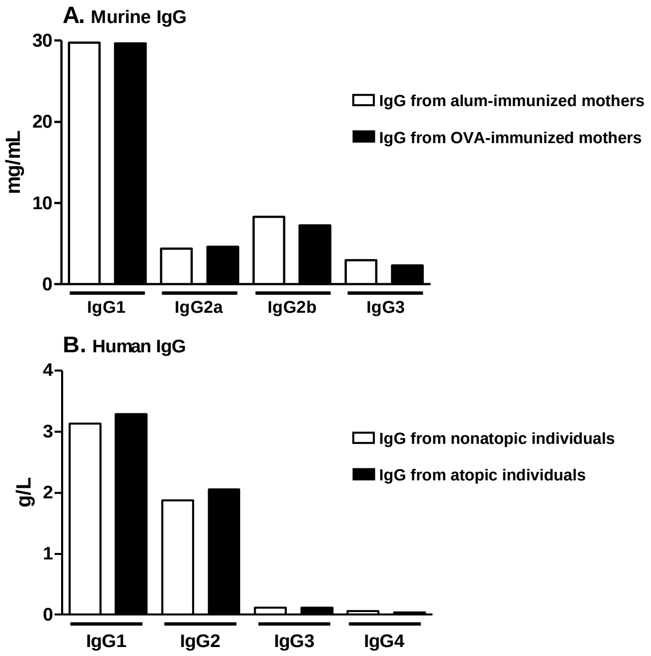

Figure S8. Murine e human IgG subclasses. Purified IgG from OVA-immunized (n=25) or Alum -immunized (n=25) females were pooled, and total IgG1, IgG2a, IgG2b, and IgG3 were evaluated by ELISA (A). IgG from atopic (n=18) or nonatopic (n=17) adults was pooled, and total IgG1, IgG2, IgG3, and IgG4 were evaluated by RID (B).
